# Job vacancies at the European Centre for Disease Prevention and Control (ECDC)

**DOI:** 10.2807/1560-7917.ES.2021.26.29.2107221

**Published:** 2021-07-22

**Authors:** Eurosurveillance editorial team

**Affiliations:** 1 European Centre for Disease Prevention and Control (ECDC), Stockholm, Sweden

The European Centre for Disease Prevention and Control (ECDC) invites applications for the following positions:

 

**Scientific Officer Surveillance, Emergency Preparedness and Response**
The application deadline expires on 19 Aug 2021.

 

**Principal Expert Epidemic Intelligence / Group Leader Epidemic Intelligence**

The application deadline expires on 26 Aug 2021.

**Expert Microbiology and Applied Molecular Surveillance**

The application deadline expires on 30 Aug 2021.

 

For more information, please visit the ECDC website: 

https://ecdc.europa.eu/en/work-us/vacancies?f%5B0%5D=deadline_date%3A1

